# Influenza Virus Infection, Interferon Response, Viral Counter-Response, and Apoptosis

**DOI:** 10.3390/v9080223

**Published:** 2017-08-12

**Authors:** Jung Min Shim, Jinhee Kim, Tanel Tenson, Ji-Young Min, Denis E. Kainov

**Affiliations:** 1 Institut Pasteur Korea, Gyeonggi-do 13488, Korea; jungmin.shim@ip-korea.org (J.M.S.); jinhee.kim@ip-korea.org (J.K.); jiyoung.min@ip-korea.org (J.-Y.M.); 2 Institute of Technology, University of Tartu, Tartu 50090, Estonia; tanel.tenson@ut.ee; 3 Department of Clinical and Molecular Medicine, Norwegian University of Science and Technology, Trondheim 7028, Norway

**Keywords:** influenza virus, apoptosis, antiviral agent, innate immunity, host response

## Abstract

Human influenza A viruses (IAVs) cause global pandemics and epidemics, which remain serious threats to public health because of the shortage of effective means of control. To combat the surge of viral outbreaks, new treatments are urgently needed. Developing new virus control modalities requires better understanding of virus-host interactions. Here, we describe how IAV infection triggers cellular apoptosis and how this process can be exploited towards the development of new therapeutics, which might be more effective than the currently available anti-influenza drugs.

## 1. Introduction

Influenza A and B viruses are common causes of seasonal epidemics. Infected individuals display mild symptoms like cough, sore throat, nasal discharge, fever, headache, and muscle pain [1]. However, the symptoms can be more severe and lead to serious complications like bronchitis and pneumonia. Globally, influenza viruses are the culprits in 3–5 million annual cases of hospitalization and 250,000–500,000 deaths [2,3].

Influenza A virus (IAV) in particular is a potential threat to global health. In contrast to influenza B virus which is only found in humans, IAV can cause pandemic outbreaks when a novel subtype emerges, typically from an animal origin [4]. In the 20th century alone, four influenza pandemics were recorded. The most severe pandemic “Spanish Flu” swept the continents in 1918–1919, affected 500 million people, and caused over 30 million deaths [5]. The most recent pandemic in 2009 emerged when the swine-origin virus, so called “Swine flu”, began to infect humans [6]. In addition, “Avian Flu” represents an ongoing threat that may result in devastating consequences if not controlled.

Anti-influenza drugs that target influenza neuraminidase (NA) have been used to prevent and treat influenza virus infections for many years. In particular, oseltamivir, zanamivir, and peramivir exert antiviral effects [7], but certain amino acid changes in NA give rise to drug-resistant IAV strains [8,9]. Due to increasing cases of drug-resistance, and thus reduced efficacy of current treatment, a critical question remains: what will be the next generation of anti-influenza drugs that is less likely to lead to a selection of drug-resistant virus variants?

Developing new virus control modalities requires better understanding of virus-host interactions. Here, we attempt to summarize our knowledge in virus-host cell interactions with a particular focus on programmed death of infected cells (apoptosis). We propose a concept of using apoptosis-inducing drugs as a new class of potential anti-influenza agents. These small molecules can facilitate apoptosis of infected cells, without affecting non-infected cells and, therefore, limit IAV replication and spread. The concept can be expanded to other viral diseases.

## 2. Influenza A Virus Structure and Replication Cycle

IAV belongs to the *Orthomyxoviridae* family [10]. Its genome is comprised of eight single-stranded viral RNA segments (vRNA) of negative polarity. Two gene segments encode pre-mRNAs that are alternatively spliced to produce nonstructural protein 1 (NS1)/nuclear export protein (NEP) and matrix M1/proton channel M2 proteins, whereas six others encode mRNAs which are translated into nucleoprotein (NP), polymerase subunit PA, PB1 or PB2, hemagglutinin (HA), and NA. Two of the six mRNAs, however, can be translated using different start/stop codons to produce PA-X/N40 and PB1-F2 [10].

In the virions, NP and three viral polymerase subunits bind to vRNA to make eight viral ribonucleoproteins (vRNPs). Eight vRNPs are surrounded by M1 and a lipid membrane, derived from the host cell. The membrane is embedded with HA, NA, and M2. NS1, NEP, PB1-F2, PA-X, and N40 are only expressed in the infected cells and not present in the virion.

IAVs are divided into subtypes based on the structure of virus surface glycoproteins HA and NA. Currently, there are 18 known subtypes of HA (H1-18) and 11 of NA (N1-11) [11]. Only a limited number of IAV subtypes including H1N1 and H3N2 are capable of infecting humans.

**Figure 1:**
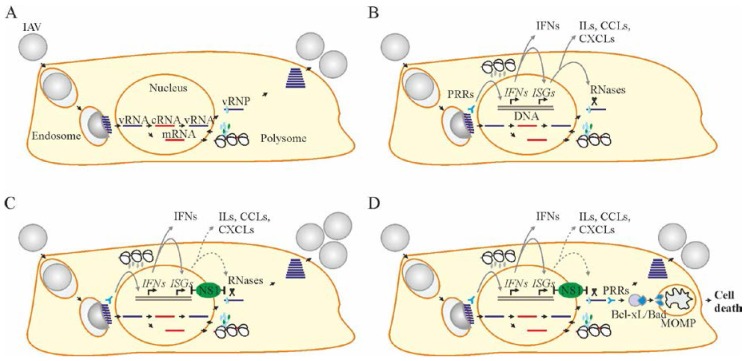
Influenza A virus (IAV) replication cycle, interferon (IFN) response, viral counter-response, and apoptosis. (**A**) IAV replication cycle consists of entry through endocytosis into the host cell and uncoating of viral ribonucleoproteins (vRNPs), import of vRNPs into the nucleus, transcription and replication of the viral genome, translation of viral proteins in the cytoplasm, assembly of vRNPs in the nucleus, export of the vRNPs from the nucleus, and assembly and budding of virions at the host cell plasma membrane. (**B**) When IAV enters the cell, pathogen recognition receptors (PRRs) sense viral RNA (vRNA) and initiate the transcription of interferon (*IFN*) genes. Once transcribed, *IFNs* mediate the expression of IFN-stimulated genes (*ISGs*) in self or, when secreted, in neighboring non-infected cells. *ISGs* encode different antiviral proteins including RNases, which degrade vRNA in infected cells. *ISGs* also encode interleukins (ILs), C-X-C and C-C motif chemokines (CXCLs and CCLs) and other cytokines to recruit immune cells to the site of infection. (**C**) IAV nonstructural protein 1 (NS1) hinders the cellular *IFN-ISG* response by binding with cellular DNA, vRNA, or other cellular factors. The viral replication cycle continues. (**D**) Apoptosis is initiated in response to a large amount of vRNA or its replication intermediates. PRRs recognize vRNA and transduce signals to anti-apoptotic B-cell lymphoma 2 (Bcl-2) proteins. Bcl-2 proteins release pro-apoptotic proteins to initiate mitochondrial outer membrane permiabilization (MoMP), ATP degradation and caspase 3 activation. This results in cell death.

The replication cycle of IAV begins when the HA bind to sialic acids on the surface of epithelial cells of the respiratory tract, dendritic cells, type II pneumocytes, alveolar macrophages, or retinal epithelial cells (Figure 1A) [12,13,14]. Viruses are internalized by endocytosis and then transported to late endosomes [15]. The acidic environment in the late endosomes facilitates HA-mediated fusion of the viral and endosomal membrane, followed by degradation of M1 and release of vRNPs in the cytoplasm [16,17]. The vRNPs enter the nucleus [18]. In the nucleus, negative-sense vRNA is transcribed into positive-sense mRNA using viral polymerase [19,20]. The polymerase snatches 5′ caps from cellular RNA and 3′ RNA is polyadenylated in order to make viral pre-mRNA. The viral proteins are translated from mRNA in the cytoplasm by ribosomes in a cap-dependent manner. Some viral proteins are imported into the nucleus to replicate vRNA. Replication of vRNA occurs in two steps: (i) synthesis of positive-sense complementary RNA (cRNA); (ii) copying of cRNA into new negative-sense vRNAs. Newly assembled vRNPs and viral proteins are transported to the apical side of the cell plasma membrane, where virions are assembled and released by NA [21].

Approximately 0.18–0.21% of amino acids in IAV proteins mutate every year due to the error-prone nature of viral polymerase [22]. Some of these mutations cause antigenic drift, which allows emerging viruses to evade host immunity developed from previous IAV infections or vaccinations. The viruses can also undergo reassortment of genetic segments to generate even greater variations and sometimes antigenic shift. The genetic shifts and drifts are potential causes of epidemic and pandemic outbreaks [10].

## 3. Cellular Factors Essential for Influenza A Virus Replication

Partly due to the simplicity of the genome, IAVs complete successful replication by relying on multiple cellular proteins [23,24,25,26,27,28,29,30]. Cellular clathrin, epsin-1 Ras-related GTPases, and COPI are important for virus dynamin-dependent endocytic uptake. Cellular vATPase acidifies the interior of late endosomes. This activates cellular serine proteases, which cleave HA and mediate fusion of viral and endosomal membranes and the release of vRNPs surrounded by M1. The aggresome formation and disassembly machinery degrades the M1 shell and uncoats vRNPs. Subsequently, cytoplasmic importins mediate nuclear import of vRNPs through the nuclear pore complex (NPC). Cellular hCLE, cyclin T1, CDK9, ANP32A, and pol II are required for vRNA transcription. PTBP1, NHP2L1, SNRP70, SF3B1, SF3A1, CLK1, UAP56, p14, and PRPF8 are necessary to splice NS1/NEP and M1/M2 pre-mRNAs. NPC, with the help of cellular NXF1, E1B-AP5, Rae1, and p15, transport viral mRNAs into the cytoplasm. In the cytoplasm, a translation apparatus translates viral mRNAs into proteins and GRSF1 stimulates this process. Subsequently, quality control of newly synthesized viral proteins is carried out by cellular chaperones and chaperonins. In addition, ISGylation, SUMOylation, and phosphorylation processes mediated by cellular machineries could modify novel viral proteins. Importins and HSP90 assist in the translocation of viral polymerase, NP, and NEP via NPC back to the nucleus where they form NEP-vRNP complexes. Crm1, HRB, hNup98, and Raf–MEK–ERK are required for transport of NEP-vRNPs into the cytoplasm through NPC. In the cytoplasm, microtubules and Rab11 bring the complexes to the plasma membrane. Newly synthesized M1, M2, HA, and NA are also transported to the plasma membrane through the trans-Golgi network with the help of COPI and Rab8. β-actin, CK2 and Rab11 are cellular proteins required for the budding and release of new virions.

IAV also actively exploits cell metabolism for the production of viral RNA, proteins, and lipids [24,26,31,32,33,34,35,36]. Free NTPs are used by viral polymerase which produces vRNA and its replication intermediates. In addition, IAV utilizes amino acids to synthesize viral proteins by hijacking the PI3K–mTor–Akt-mediated autophagy. Virus assembly and budding depends on lipid metabolism (including fatty acid biosynthesis, phospholipid metabolism, de novo synthesis of cholesterol). Finally, virus replication is sensitive to the cellular redox state, which is essential for maturation of HA and for the quality of released viral particles. These are only a few examples of cellular factors essential for virus replication.

## 4. Cellular Factors that Limit Virus Replication and Spread

Apart from cellular factors that support viral replication, there are dozens of those which restrict this process. When IAV enters the cells, stimulus-specific signals are transduced along the interferon signaling pathway to activate antiviral responses (Figure 1B) [37]. Pattern recognition receptors (PRRs), such as TLR3, TLR7, IRF7, MDA5, and RIGI sense incoming viruses and activate transcription of interferon (IFN) genes, such as *IFNB1*, *IL28A*, *IL29*, *IL28B*, *IFNW1*, *IFNA7*, *IFNA14*, *IFNA10*, *IFNA13*, *IFNA16*, *IFNA8*, *IFNA1*, *IFNG*, *IFNA2*, and *IFNA21* [38]. IFNs launch the expression of IFN-stimulated genes (*ISGs*) in infected cells as well as in nearby non-infected cells, protecting them from potential viral invasion (Figure S1) [39,40,41].

**Figure 2:**
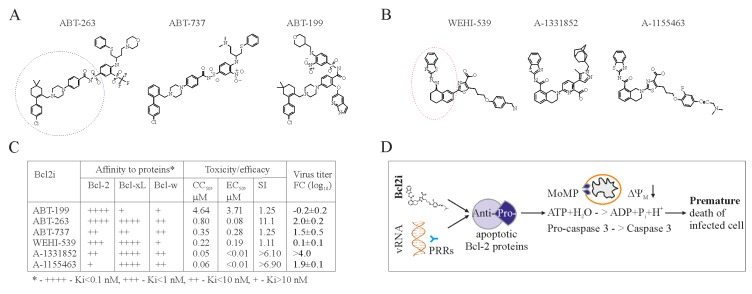
Bcl-2 inhibitors (Bcl2i) facilitate Bcl-2-dependent apoptosis in cells containing viral RNA. (**A**,**B**) Structures of ABT-263, ABT-737, ABT-199, WEHI-539, A-1331852, and A-1155463 revealed that these molecules fall into two distinct classes. Core structures are highlighted. (**C**) Table showing Bcl2i antiviral activities and affinities for three Bcl-2 proteins. “+” indicates inhibitory effect. Increased inhibition is marked by a higher “+” designation. (**D**) Schematic diagram showing how chemical inhibitors of Bcl-2 proteins induce premature death of cells containing viral nucleic acids. Bcl: B-cell lymphoma; CC_50_: half-maximum cytotoxic concentration; EC_50_: half-maximum efficacy concentration; SI: selectivity index; FC: fold-change; PRRs: pattern recognition receptors.

The ISGs encode a variety of antiviral proteins with diverse modes of action. These include IFITM1 and SAMD9, which prevent fusion between viral and endosome membranes; HERC5, HERC6, USP18, ISG15, TRIM22, and ISG20, which mark viral proteins for degradation and, thereby, mediate vRNA uncoating; IFIT1, IFIT2, OASL, IRF7, DDX60, DDX58/RIG-I, IFIH1/MDA5, and EIF2AK2/PKR, which recognize vRNA, and OAS1, OAS2, and OAS3 which degrade vRNA; ZBP1, PARP1, PARP9, PARP14, and PRIC285, which inhibit transcription and translation of vRNA and activating expression of cellular antiviral genes; lipid raft-disturbing factor RSAD2 which prevents coating of vRNPs with host membrane; and cholesterol-depleting factor IFITM3 which inactivates budding viruses (Figure 2B) [42,43,44,45,46,47]. ISGs also encode IFI27 and XAF1 for regulation of apoptosis; IDO, COX2, and CH25H for production of neuro- and immuno-modulators; cytokines and chemokines for activation and recruitment of immune cells to the site of infection; MX1, MX2, GBP1, GBP2, GBP3, GBP5, IFI44, GMPR, and NT5C3 for GTP catabolism and cytokine processing; STAT1 for amplification of autocrine *ISG* expression, as well as many other antiviral factors. As a result, *ISG* products can inhibit viral replication in infected cells, alert non-infected cells for potential infections, attract immune cells, and trigger an alarm in the central nervous system about the ongoing infection.

In counter-response to cellular IFNs, IAV utilizes non-structural protein NS1 (Figure 1C) [48]. NS1 is produced within a few hours of infection [49]. NS1 can block the transcription of innate antiviral genes by directly binding with cellular DNA [50]. In addition, NS1 interacts with vRNA and its replication intermediates to prevent its recognition by cellular PRRs and RNAses [51,52,53,54]. It can also bind TRIM25, ISG15, GBP1, and other *ISG* products to inhibit their functions at transcriptional, post-transcriptional, translational, and post-translational stages [55]. Thus, the levels of innate immune mediators are regulated by viruses to ensure IAV replication and to avoid excessive IFN responses, which are often associated with severe disease [56,57].

## 5. Apoptosis Is a Cellular Process That Restricts Virus Replication and Spread

When the IFN responses fail to control IAV replication, cells may activate a secondary antiviral response via programmed death called apoptosis (Figure 1D). This is particularly important when IAV escapes the IFN responses through the action of NS1. During this process, PRRs, including RIG-I, MDA5, PKR (encoded by ISGs: *IFIH1*, *DDX58*, and *EIF2AK2*), recognize accumulating vRNA and activate apoptotic machinery that directs the fate of IAV-infected cells [58]. The anti-apoptotic (Bcl-2, Bcl-xL, and Bcl-w) and pro-apoptotic (Bax, Bak, Bad, Bim, Bid, Puma, and Noxa) Bcl-2 proteins associate or dissociate to start a cascade of reactions resulting first in mitochondria membrane permeabilization (MoMP). This is followed by cytochrome c release, apoptosome activation, ATP degradation, and eventually cell death [59,60,61,62]. As the initial trigger of this process, the concentration of vRNA is, therefore, a critical rate-limiting factor. Alternatively, if the viral load is high enough, apoptosis could be initiated during virus entry.

All Bcl-2 proteins contain Bcl2-homology 3 (BH3) domains, which are essential for their protein-protein interactions and functions [63]. Cellular proteins including UACA, PAWR, FLII, Trim21, IMMT, 14-3-3, EFHD2, DHX9, DDX3, NLRP3, and LRRFIP2 as well as viral factors M2, PB1-F2, NS1, HA, and NP may stabilize or disrupt the interactions of BH3-domain proteins in infected cells [62,64,65,66]. However, further studies are required to verify their specific functions in apoptosis.

## 6. Apoptosis-Inducing Small Molecules

Bcl-2 dependent apoptosis represents a potential target for antiviral drug development. In particular, anticancer Bcl-2 inhibitors (Bcl2i) may be repurposed to treat viral diseases. The first anticancer Bcl-2 inhibitor, ABT-737, was engineered based on the structure of Bad bound to Bcl-xL in order to mimic Bad BH3-peptide [67,68]. Several derivatives have been developed to have improved pharmacokinetic properties, and the resulting product, ABT-263, is currently in clinical trials, and ABT-199 is approved to treat multiple lymphoid malignancies (Figure 2A) [63,69,70,71]. Another group of Bcl2i with anticancer properties was discovered using high-throughput screening [72]. This includes WEHI-539 and its derivatives, A-1331852, and A-1155463 (Figure 2B). Also, other Bcl-2 inhibitors (such as TW-37, gossypol, UMI-77, A-1210477 and BDA-366) that are structurally distinct from ABT-737 and WEHI-539 have been developed. All these compounds have different affinities for Bcl-2 proteins [58].

Importantly, ABT-737, ABT-263, ABT-199, WEHI-539, A-1331852, and A-1155463, but not TW-37, gossypol, UMI-77, A-1210477, and BDA-366, can universally induce premature death of IAV-infected cells at concentrations not toxic for non-infected cells (Figure 2C) [62]. However, only ABT-263, A-1331852, and A-1155463 could effectively limit viral replication and spread (unpublished data [73]).

We propose a model for this effect in Figure 2D. PRRs recognize vRNA or its replication intermediates and send signals to anti-apoptotic Bcl-xL. Bcl-xL releases its pro-apoptotic partners to initiate MoMP, ATP degradation, and caspase-3 activation. This results in cell death. ABT-263, A-1331852, or A-1155463 act synergistically with viral RNA and thereby facilitate the cell death.

ABT-263, unlike A-1155463, causes irreversible thrombocytopenia [74,75], which makes A-1155463 a better candidate for antiviral testing in animals. Moreover, half-maximum efficacy concentration (EC_50_) for A-1155463 is lower than that for ABT-263. In addition, half-maximum cytotoxic concentration (CC_50_) value of A-1155463 is higher than that of A-1331852, whereas EC_50_ of both are lower than that for ABT-263 (unpublished data [73]). Thus, A-1155463 could represent an antiviral lead candidate, which would reinforce the necessary therapeutic arsenal for the treatment of influenza and perhaps other viral diseases.

## 7. Accelerating Apoptosis of Infected Cells: A Novel Antiviral Strategy

**Figure 3:**
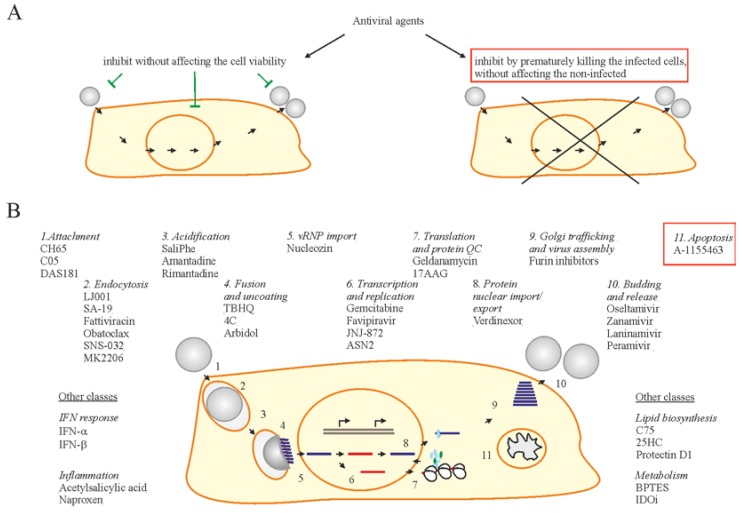
Two strategies of antiviral drug development. (**A**) One strategy is focused on discovery of antivirals to inhibit viral infection without affecting the viability of infected cells, whereas another exploits small molecules to inhibit viral replication by specifically killing only the virus-infected cells. (**B**) Examples of existing and emerging anti-IAV drugs. Existing and emerging drugs that target certain stages of virus replication cycle are shown. Bcl2 inhibitors (Bcl2i) are shown in a red box.

The typical approach in antiviral drug discovery has been to identify virus inhibitors that target various stages of virus replication and to preserve infected cells from death (Figure 3) [23,24,30,44,76,77,78,79,80,81,82,83]. Examples of such antiviral drugs are DAS181, JNJ872, ribavirin, verdinexor, CH65, C05, SaliPhe, nucleozin, geldanamycin, 17-AAG, LJ001, SA-19, fattiviracin, TBHQ, 4C, gemcitabine, ASN2, bortezamib, carfilzomib, C75, 25HC, SNS-032, and MK2206 (Figure 3) [23,24,44,79,80,81,82,83,84]. As an alternative to the traditional method, there is the use of Bcl2i. The Bcl2i selectively causes apoptosis in only virus infected cells, leaving virus-free cells intact. Therefore, Bcl2i represents a novel class of antiviral compounds with potential that is worth exploring.

However, Bcl2i must be used as a prophylactic rather than a therapeutic drug because of the following issues. Although the induction of apoptosis has been shown to be selective for infected cells in vitro, inhibition of Bcl2 proteins may have off-target effects in vivo [74,75]. Our preliminary results also indicate that treatment with Bcl2i of IAV-infected mice may affect cytokine expression and, therefore, may prevent development of innate and adaptive immune responses [62]. In addition, Bcl2i may have adverse effects in acute virus infection. The viral dose is likely to be high, infecting a large number of cells. Inducing apoptosis may result in extensive tissue damage in this case.

## 8. Conclusions

Cellular antiviral responses including IFN response and apoptosis are employed in order to inhibit virus replication and spread. IAV has evolved to gain mechanisms to disconcert these responses to ensure its replication. Based on our knowledge on host-virus interaction, we can explore ways to develop pharmacological interventions to control IAV infections. In particular, our advance in understanding apoptosis has shown potential in developing apoptosis-inducing molecules as antiviral drugs against flu. A-1155463 could serve as a lead compound in this process. Prophylactic treatment with A-1155463 may prevent development of severe disease. Successful prevention of flu using Bcl2i could provide an alternative therapeutic option for IAV, against which current treatment is limited. Having wider treatment options could reduce the use of drugs targeting virus proteins, and thus slow down the rise of drug-resistant virus strain through evolutionary selection pressure. Timely use of Bcl2i may also reduce the use of antibiotics, which are utilized for treatment of secondary bacterial infections. This will limit the development of emerging antibiotic-resistant bacteria. Exploring a new class of antiviral drugs is crucial, and further investigations on the antiviral properties of Bcl2i could lead to development of new drugs to prevent other viral diseases, associated with HIV, ZIKV, HBV, and VZV (29–31).

## Supplementary Materials

The following are available online at http://www.mdpi.com/1999-4915/9/8/223/s1. Figure S1. IAV infection and interferon response in human macrophages and RPE cells.

## Abbreviations

vATPase: vacuolar ATPase; hCLE: human homolog of chicken CLE; CDK9: cyclin-dependent kinase 9; ANP32A: acidic leucine-rich nuclear phosphoprotein 32 family member A; pol II: DNA-directed RNA polymerase 2; PTBP1: polypyrimidine tract-binding protein 1; NHP2L1: NHP2-like protein 1; SNRP70: small nuclear ribonucleoprotein U1 subunit 70; SF3B1: splicing factor 3B subunit 1; SF3A1: splicing factor 3A subunit 1; CLK1: dual specificity protein kinase 1; UAP56: spliceosome RNA helicase DDX39B ; p14: pre-mRNA branch site protein p14; PRPF8: pre-mRNA-processing-splicing factor 8; NXF1: nuclear RNA export factor 1; E1B-AP5: heterogeneous nuclear ribonucleoprotein U-like protein 1; Rae1: mRNA export factor 1; p15: mRNA export factor; GRSF1: G-rich sequence factor 1; Crm1: chromosome region maintenance 1; HRB: human immunodeficiency virus rev-binding; hNup98: human nucleoporin 98; Raf: Raf proto-oncogene serine/threonine-protein kinase; MEK: dual specificity mitogen-activated protein kinase kinase 1; Erk: extracellular signal–regulated kinases; Rab11: Ras-related protein 11; COPI: coatomer 1 vesicular transport complex; Rab8: Ras-related protein 8; CK2: casein kinase 2; PI3K: phosphatidylinositol 3-kinase; mTor: mechanistic target of rapamycin; Akt: protein kinase B; TLR3: Toll-like receptor 3; TLR7: Toll-like receptor 7; IRF7: Interferon regulatory factor 7; MDA5: interferon-induced helicase C domain-containing protein 1; RIGI: probable ATP-dependent RNA helicase DDX58; HERC5: E3 ISG15--protein ligase 5; HERC6: E3 ISG15--protein ligase 6; USP18: Ubl carboxyl-terminal hydrolase 18; ISG15: ubiquitin-like protein 15; TRIM22: E3 ubiquitin-protein ligase 22; ISG20: ubiquitin-like protein 22; IFIT1: interferon-induced protein with tetratricopeptide repeats 1; IFIT2: interferon-induced protein with tetratricopeptide repeats 2; OASL: 2′-5′-oligoadenylate synthase-like protein; DDX60: probable ATP-dependent RNA helicase 60; EIF2AK2: Interferon-induced, double-stranded RNA-activated protein kinase; OAS1: 2′-5′-oligoadenylate synthase 1; OAS2: 2′-5′-oligoadenylate synthase 2; OAS3: 2′-5′-oligoadenylate synthase 3; ZBP1: Z-DNA-binding protein 1; PARP1: poly [ADP-ribose] polymerase 1; PARP9: poly [ADP-ribose] polymerase 9; PARP14: poly [ADP-ribose] polymerase 14; PRIC285: helicase with zinc finger domain 2; RSAD2: radical S-adenosyl methionine domain-containing protein 2; IDO: indoleamine 2,3-dioxygenase 1; COX2: Prostaglandin G/H synthase 2; CH25H: Cholesterol 25-hydroxylase; MX1: interferon-induced GTP-binding protein 1; MX2: interferon-induced GTP-binding protein 2; GBP1: guanylate-binding protein 1; GBP2: guanylate-binding protein 2; GBP3: guanylate-binding protein 3; GBP5: guanylate-binding protein 5; IFI44: interferon-induced protein 44; GMPR: GMP reductase 2; NT5C3: cytosolic 5'-nucleotidase 3A; GTP: guanosine triphosphate; Bax: apoptosis regulator BAX; Bak: Bcl-2 homologous antagonist/killer; Bad: Bcl2-associated agonist of cell death; Bim: Bcl-2-like protein 11; Bid: BH3-interacting domain death agonist; Puma: Bcl-2-binding component 3; Noxa: phorbol-12-myristate-13-acetate-induced protein 1; 25HC: 25-Hydroxycholesterol; HIV: human immunodeficiency virus; ZIKV: Zika virus; HBV: hepatitis B virus; VZV: Varicella zoster virus.
