# Supplementary material for: Influenza Virus Infection, Interferon Response, Viral Counter-Response, and Apoptosis
Source: Viruses. 2017 Aug 12;9(8):223. doi: 10.3390/v9080223 (PMC5580480; doi:10.3390/v9080223)
Supplement: Supplementary file 1 [file viruses-09-00223-s001.docx]

**Supplementary material for “Influenza virus infection, interferon response, viral counter-response and apoptosis” by Shim et al.**

**
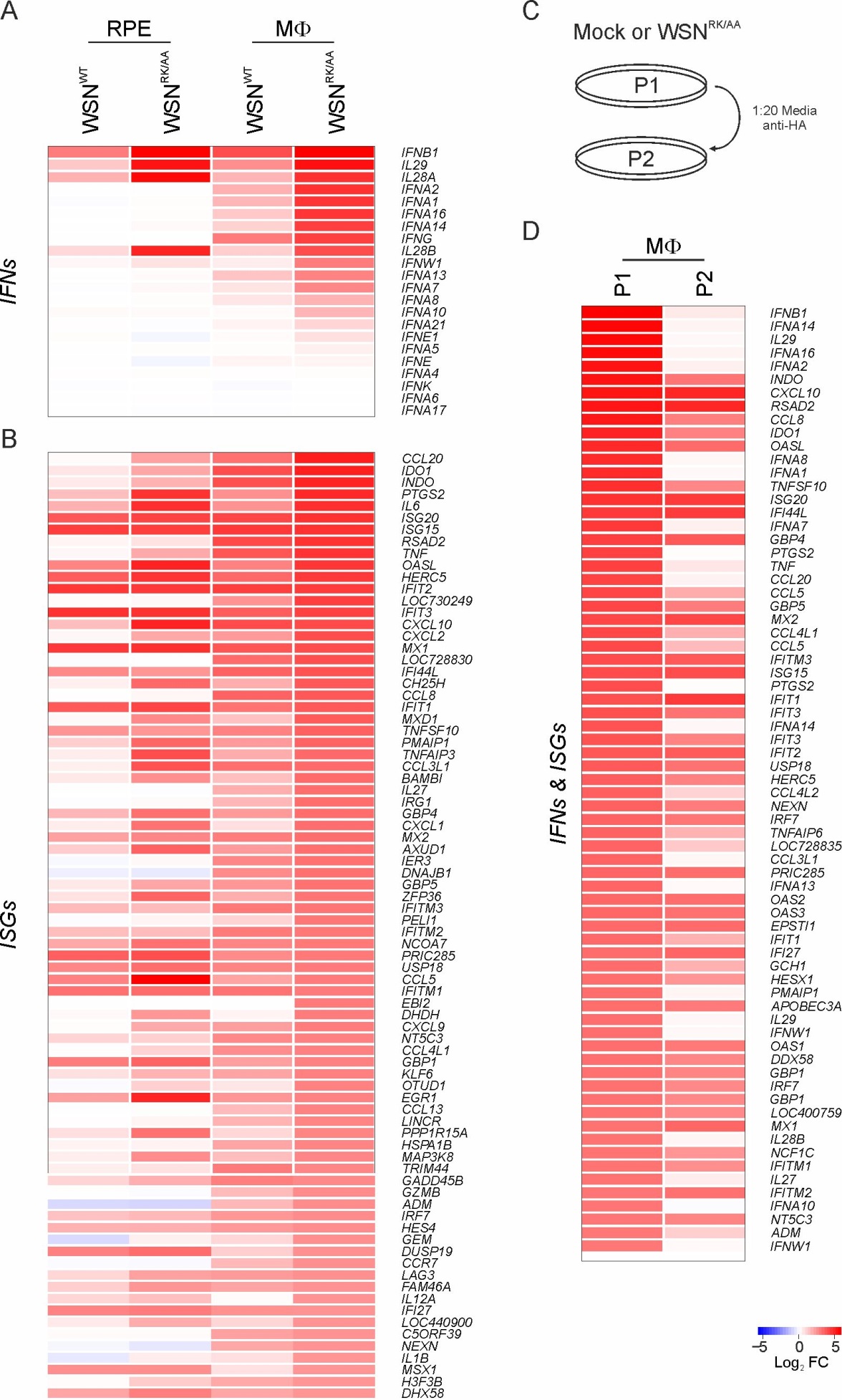
**

**Figure S1.** IAV infection and interferon (IFN) response in human macrophages and RPE cells. (**A**, **B**) Transcriptional response of macrophages and RPE cells to infection with wild-type WSN strain or its mutant version, which is unable to suppress transcription of *IFNs* and interferon-stimulated genes (*ISGs*). Cells were mock-, WSN^WT^- or WSN^RK/AA^ –infected (multiplicity of infection 3). After 8 h of infection total RNA was extracted from the cells and gene expression was profiled using microarrays. Statistically significant (*p* < 0.05) differences in *IFN* and *ISG* expression between virus- and mock-infected cells are shown on a heatmap. Each cell is colored according to the average of the log2-transformed and quantile-normalized expression values of the triplicate samples with the average of mock controls subtracted. (**C**, **D**) Response of macrophages infected with IAV or treated with IFNs, produced by infected IAV-infected macrophages. Macrophages (P1) were infected with WSN^WT^ (multiplicity of infection 3) or mock. After 1 h, the media were changed to a fresh one. After 24 h, the media were collected, treated with anti-hemagglutinin antibodies and diluted with fresh medium. The media were applied to macrophages (P2) from the same donor. RNA was extracted from P1 and P2 cells after 8-h post-infection and subjected to genome-wide transcription profiling. A heatmap map of *IFNs* and *ISGs* is shown. Rows represent gene symbols, columns represent treatments. Each cell is coloured according to the log2-transformed and quantile-normalized expression values of the samples, expressed as fold-change relative to the average of mock controls.
